# Correlational research on facial and clinical characteristics of adolescents with obsessive-compulsive disorder

**DOI:** 10.1186/s12888-021-03612-5

**Published:** 2021-12-11

**Authors:** Yan-Rong Wang, Shao-hua Chang, Xiao-Min Ma, Ji-Ying Li, Rui-Xia Zhang, Jian-Qun Fang

**Affiliations:** 1 Mental Health Centre, General Hospital of Ningxia Medical University, No. 804 South Shengli Street, Yinchuan, 750004 Ningxia China; 2 Ningxia Medical University, No.1106 South Shengli Street, Yinchuan, 750004 China; 3 Ning An Hospital, Ningxia Mental Health Center, Yinchuan, 750004 China

**Keywords:** Adolescent, Obsessive-compulsive disorder, Neurodevelopment, Facial characteristics

## Abstract

**Background:**

The neurodevelopmental model of obsessive-compulsive disorder (OCD) suggests that the neurodevelopmental changes in the ventral striatal circuit of the prefrontal lobe are associated with the initial symptoms of OCD. Facial morphology is one of the most consistent anatomical phenotypes of neurodevelopmental disorders, which can reflect brain structure and function. Facial deformity, an easily measured index of brain malformation, can reflect abnormal brain structure and function. Therefore, this study aims to explore the relationship between clinical features and neurodevelopment of adolescents with OCD through facial morphology.

**Methods:**

The enrolled study sample comprised 40 adolescents diagnosed with OCD using the Obsessive Compulsive Inventory-Child Version (OCI-CV) and 38 healthy controls (HCs). Facial photos, 21 facial diameters, and 9 facial angles were collected using image software.

**Results:**

In males, lower lip red height was significantly lower in OCD patients than in HCs (*P* < 0.025); no significant differences were observed in other facial indicators (all *P* > 0.025). In females, the nasolabial angle was smaller in OCD patients than in HCs (*P* < 0.025); no significant differences were observed in other facial indicators (all *P* > 0.025). The difference in lower lip red height between the OCD group and HC group was positively correlated with neutralizing symptoms (r = 0.401, *P* < 0.05).

**Conclusions:**

Male OCD patients had a thinner lower lip and female OCD patients had smaller nasolabial angles. The facial features of adolescents with OCD were positively correlated with lower lip redness and neutralizing symptoms.

## Background

Research over the last two decades has identified obsessive–compulsive disorder (OCD) in children and adolescents as one of the most common psychiatric illnesses affecting youth. Epidemiological studies have reported a lifetime prevalence of 1–3% in pediatric populations [1] and 30–50% of adult patients develop OCD during childhood and adolescence [2]. OCD in youth is typically a chronic and debilitating disorder [3], and the morbid dysfunction caused by OCD commencing in youth may interfere with the trajectory of normal development during critical periods. This may impede the consolidation of processes such as identity and personality formation, social and educational mastery, and future orientation [3]. Given the high prevalence, morbidity, and functional impairments associated with OCD in children, early detection is crucial.

Children with OCD often present with neurodevelopmental disorders such as tic disorder and attention deficit hyperactivity disorder [4], which are underpinned by a common biological basis. The neurodevelopmental model of OCD, proposed by Rosenberg and Keshavan, theorizes that neurodevelopmental changes in the ventral striatal circuit of the prefrontal lobe are associated with the initial symptoms of OCD [5]. Growing neuroimaging evidence has highlighted asymmetries and subtle changes in subcortical structures (e.g., thalamus and globus pallidus) in children with OCD, but these changes are absent in adult OCD patients [6]. A recent genomic study of mental diseases including OCD, autism, attention deficit hyperactivity disorder, and tic disorder demonstrated that pleiotropic risk sites are enriched in genes related to neurogenesis and neurodevelopment, and are expressed in the second trimester peak [7]. These findings support the neurodevelopmental hypothesis of OCD in children. However, the neurodevelopmental origin and potential causes of OCD have not been elucidated.

Minor physical anomalies (MPAs) refer to subtle morphological abnormalities of the craniofacial region and limbs [8]. These abnormalities do not result in obvious cosmetic or functional sequelae. MPAs are markers of abnormal fetal morphogenesis in the early or middle trimester of pregnancy and originate from the ectoderm alongside the fetal brain [9, 10]. As MPAs are affected by genetic and prenatal factors, they may be used as neurodevelopmental indicators [8]. MPAs include minor deformities and phenotypic variations [11, 12], and are stable over time. Minor deformities are underpinned by qualitative defects after organogenesis during embryogenesis. Phenotypic variation refers to the quantitative defects that occur after organogenesis and are equivalent to the variation in normal human body measurements [13]. Therefore, screening for MPAs can be achieved via physical measurements [12]. Previous studies have suggested that the incidence of MPAs is higher in patients with neurodevelopmental disorders such as schizophrenia, autism, and Tourette’s syndrome than healthy controls (HC) [12, 14–16]. However, there is currently a paucity of studies on MPAs in children with OCD.

Abnormal craniofacial morphology in MPAs is one of the most consistent anatomical phenotypes of neurodevelopmental disorders [17]. Atypical facial features are observed in many developmental disorders, such as 22q11.2 deletion syndrome and fetal alcohol syndrome [18, 19]. Facial morphological abnormalities vary from subtle to severe. During embryonic development, brain and facial tissues both originate from the neuroectoderm and reciprocally affect their development. Genetic or environmental events that interfere with early fetal development will result in morphological abnormalities [20]. If these deformities are sufficiently prominent, they can be qualitatively identified and classified by inspection, which is currently practiced in clinical genetics and pediatrics [21]. If abnormal facial morphology is not evident, traditional anthropometric techniques can be used to quantify and grade this morphological disorder. Based on standard anatomical landmarks of individual facial features, facial abnormalities can be measured using manual or conventional two-dimensional photographs [22], and biological significance can be determined based on morphological assessments. Facial deformity is the most easily measured index of brain malformation, which can reflect abnormal brain structure and function via facial abnormalities. Facial morphology is largely determined by genetic factors [23]. Technological advancements in facial morphological measurements using abnormal facial features to bridge clinical phenotypes and genotypes will enable the identification of genes associated with OCD. Identification of facial abnormalities using facial recognition technology [24] may facilitate early screening and auxiliary diagnosis of OCD. Based on the neurodevelopmental hypothesis of OCD in children and adolescents, this study selects facial morphological features as the observational indicators of neurodevelopment, and explores the correlation between facial features and clinical symptoms of adolescents with OCD by measuring the dimensions of the subjects’ facial photos.

The main hypothesis of the current study was that the facial morphological features are different between OCD adolescents and healthy adolescents. A secondary hypothesis was that gender affects the difference related to facial morphological features between OCD adolescents and healthy adolescents. In addition, we also hypothesized that the facial morphological features are related to clinical features of OCD.

## Methods

### Participants

Participants were 15- to 17-year-old students studying at two high schools in Ningxia Hui Autonomous Region, China. In the recruiting period, a total of 2400 students of two high school were approached. The Obsessive Compulsive Inventory Child Version (OCI-CV) was used as a screening tool for OCD. Participants who scored more than 20 points in OCI-CV (high-risk obsessive-compulsive symptoms group) and those who scored 0 points (non-obsessive-compulsive symptoms group) were further diagnosed using the MINI interview scale and Yale-Brown Obsessive-Compulsive Scale (YBOCS) by two attending psychiatrists. YBOCS ≥16 points and diagnosis of OCD using the MINI interview confirmed the OCD patients and healthy controls (HC). Of the 2400 students, a total of 98 adolescents were diagnosed with OCD according to the MINI and YBOCS. Eight were excluded due to being diagnosed with other mental illnesses or having used antipsychotics. Thirty-six were excluded due to their body index not being within the normal range. Fourteen were excluded due to having had facial plastic surgery or orthodontics. A final sample of 40 OCD patients were recruited. After matching with the OCD patients in age and sex, 38 HC out of 120 adolescents in the group without obsessive-compulsive symptoms (OCI-CV = 0) were recruited.

To be eligible for participation in the study, participants were required to fulfill the following inclusion criteria: (1) aged 15–17 years old, met the DSM-5 diagnostic criteria for OCD [25] and had a YBOCS score ≥ 16 points; (2) first onset and were not treated with serotonin reuptake inhibitors or other psychotropic drugs; (3) obsessive-compulsive symptoms were not secondary to other mental and/or physical diseases; (4) no color vision weakness or blindness; and (5) were right-handed. Participants were excluded from participation if they had: (1) a previous history of depression, panic disorder and/or schizophrenia; (2) severe brain diseases or unstable physical diseases; (3) alcohol or other substance dependence; (4) neurological or hormonal diseases; (4) mental retardation precluded cooperation in experiments; (5) serious lack of dentition, facial plastic surgery, orthodontic correction, history of surgery, and/or history of trauma; and (6) overweight or underweight.

### Power analysis

According to a previous meta-analysis, the effect size difference between OCD patients and HC in recognizing facial displays of emotion fell within a medium range of Cohen’s effect size rules [26]. To reach the medium effect size with Cohen’s *d* = 0.5, with a two-sided test *α* = 0.05 and 1-*β* = 0.95, a sample size of *N* = 54 is needed. Considering a two-group matched-subjects design, the sample size for one group is 27. The present study collected 40 OCD patients and 38 HC, which is larger than the required sample size. This suggested an adequate statistical power in the current study.

### Procedures

Participants completed all measurements via face-to-face surveys, which were conducted by research staff who received rigorous training prior to fieldwork. The study was approved by the Ethics Committee of the General Hospital of Ningxia Medical University (no.2018–131). Informed consent and assent were obtained from all participants included in the study.

### Measures

#### Assessment of obsessive-compulsive symptoms

##### The obsessive compulsive inventory-child version (OCI-CV)

The OCI-CV was compiled by Foa et al. [27] to measure obsessive-compulsive symptoms in children and adolescents aged 7–17 years. The OCI-CV comprises 21 items and uses a 3-point Likert-type scale ranging from 0 (never) to 2 (always). The score ranges from 0 to 42 points and includes six dimensions: obsessing, hoarding, washing, ordering, neutralizing and doubting/checking. The total scores of each dimension were added, with higher scores indicating more severe obsessive-compulsive symptoms. The Chinese version revised by Xing et al. [28] has demonstrated good reliability and validity in adolescents.

#### The Yale Brown obsessive compulsive scale (YBOCS)

The YBOCS was compiled by Goodman et al. [29] in 1989 to assess the severity of obsessive-compulsive symptoms in patients with OCD. The scale comprises of 10 items used to assess the severity of obsessive thinking and behavior. Each item uses a five-level scoring method of 0–4 points. The total score of the scale is 0–40. The scores of the obsessive thinking and compulsive behavior subscales range from 0 to 20, with higher scores indicating more serious obsessive-compulsive symptoms. Mild, moderate, severe, and extremely severe obsessive-compulsive symptoms are indicated by total scores of 8–15, 16–23, 24–31, and 32–40, respectively. The Chinese version has been demonstrated to have good structural and content validity [30] and was used to assess the severity of obsessive-compulsive symptoms in OCD patients in this study.

### Facial soft tissue measurement method: two-dimensional photo measurements

#### Filming locations

Two fixed classrooms in the school were selected for measurements. The classrooms were required to be quiet and bright, with suitable temperature and good lighting. The sitting positions and photo locations of participants were fixed.

#### Posture

For orthographic photographs, participants adopted a natural head position, staring ahead with their heads on the Frankfurt plane. For the positive and lateral positions, the sagittal plane of the participant’s head was positioned parallel to the plane of the background screen, and the Frankfurt plane was parallel to the ground. The facial muscles relaxed naturally, and the hair was combed behind the ears to expose the forehead and ears. Tooth occlusion was positioned at the largest occlusion.

#### Photographic tools and photography methods

A Canon EOS700D single-lens reflex digital camera with 18 million pixels was selected. A blue background curtain marked with measurement signs was hung on the classroom wall. Participants were seated in a fixed position. The camera was fixed on a tripod and kept parallel to the ground. The object distance (distance from the lens to the tip of the nose) was 150 cm. The lens center was aligned with the tip of the nose. The shutter speed was 1/60 s. The focal length was 72 mm. The aperture was F3.5. The horizontal and vertical lines of the lens were required to overlap the eye-ear plane and center line of the face, respectively. The shutter was pressed at this time to obtain a facial image. Frontal and lateral photographs were obtained for each participant.

#### Photo measurement method

Anteroposterior and lateral photo data of the two groups were imported into a computer for storage. Digimizer professional medical measurement image software was used to perform fixed-point measurements of the photographs within a period.

#### Determination of facial soft tissue measurement indexes

Using the facial morphometric method established by Deutsch and Farkas [22], 21 facial diameters (see Table 1), and 9 facial angles were measured (see Table 2).

**Table 1:** The facial diameter

Facial diameter	abbreviation	Measurement of facial diameter
Minimum forehead width (upper face width)	ft-ft	The straight-line distance between left and right frontal and temporal points
Face width (central face width)	zy-zy	The straight-line distance between the zygomatic points on the left and right sides
Interocular width(intercanthal width)	en-en	The straight-line distance between the inner corners of the left and right eyes
Eye cleft width (left and right)	en-ex	The horizontal distance from the inner canthal point to the perpendicular to the ipsilateral outer canthal point
Eye cleft height (left and right)	ps-pi	The distance between the midpoints of the upper and lower eyelid margins
Mandibular angle width (lower face width)	go-go	The straight-line distance between the left and right mandibular corner points
Nasal width	al-al	The straight-line distance between the left and right nose points
Philtrum width	ms-ms	The distance between midpoints of cristae
Philtrum length	sn-ls	The distance from the lower nose to upper lip
Oral fissure width	ch-ch	The straight-line distance between the left and right corners
Lip height	ls-li	The straight-line distance between the upper lip point and lower lip
Distance between the high points of the lip arch	cp-cp	The distance between the high points of the lip arch
Forehead distance	tr-n	The projection distance from the hairline point to root point of the nose
Nasal height	n-sn	The straight-line distance from the base of the nose to point below the nose
Nasal length	n-prn	The distance from the base of the nose to tip of the nose
Full lip height	sn-sto	The straight-line distance from the point of the nose to point of the cleft
Upper lip height	ls-sto	The vertical distance between the midpoint of the upper lip and cleft point
Lower lip high	sto-li	The vertical distance between the midpoint of the lower lip and cleft point
Nose high profile	n-sn	The linear distance from the base of the nose to point below the nose from the side
Long nose profile	n-prn	The distance from the base of the nose to tip of the nose from the side
Jaw height	sto-gn	The distance from the point of the mouth to submental point

**Table 2:** The facial angle

Facial angle	Abbreviation	Measurement of facial diameter
Full surface coign	∠g-prn-pg’	The angle between the base of the nose, tip of the nose, and front of the chin reflects the protrusion of the face including the nose
Surface coign	∠g-sn-pg’	The angle between the base of the nose, point of the nose, and point of the front of the chin reflects the protrusion of the soft tissue profile
Upper lip and chin process angle	∠ula-ns-pos	The angle between the protrusion of the upper lip and base of the nose, and between the base of the nose and anterior point of the soft tissue, indicates the protrusion of the upper lip relative to the face
Lower lip and chin process angle	∠lla-ns-pos	The angle between the protrusion of the upper lip and base of the nose, and between the base of the nose and anterior point of the soft tissue, indicates the protrusion of the upper lip relative to the face
Nasofrontal angle	∠g-ns-prn	The dorsal line of the nose intersecting the inclined plane from the forehead to the root of the nose reflects the degree of depression of the root of the nose or degree of protrusion of the forehead
Angle of nasal process	∠n-prn-sn	The included angle of the line connecting the base of the nose-tip point-under-nose point is the angle between the dorsal line of the nose and the line of the columella. Reflect the protrusion of the nose itself
Chin Angle of upper and lower lip	∠ula-ns-lla	The angle between the protruding point of the upper lip and the base of the nose, and the protruding point of the base of the nose and the protruding point of the lower lip, indicating the relative position of the upper and lower lips
Nasolabial angle	∠cm-sn-ula	The intersection angle of the line between the columella point and subnasal point, and the line between the subnasal point and upper lip protrusion, reflects the morphological changes and forward and backward positions of the nose and upper lip
Chin lip groove angle	∠pos-sl-li	The angle between the point of the lower lip, point on the chin, and the front of the chin indicates the degree of lower lip protrusion relative to the chin

### Statistical analyses

All analyses were conducted using SPSS software (version 21.0, IBM Corp., Armonk, NY, USA). All reported *p*-values are two-tailed. The level of statistical significance was set at *P* < .05. Frequencies/percentages and means/standard deviations describe the distributions of participants according to demographic characteristics. T-test and chi-square test were used to compare two groups. Data were tested for normality, the correlation between facial and clinical features was analyzed by Pearson correlation. Bonferroni corrections were conducted for analysis of gender difference and *P* < .025 was considered as significant level. Cohen’s *d* was calculated as effect size.

## Results

### Sample characteristics

The study consisted of 78 adolescents (*n* = 40 in the OCD group and *n* = 38 in the HC group) aged 15–17 years old (M = 16.25, SD = 0.87). Table 3 presents detailed information on demographics (sex, age, BMI, and years of education) and YBOCS score data. The average YBOCS score of OCD patients (M = 24.23, SD = 5.45) was higher than that of HC (M = 1.71, SD = 2.19).

**Table 3:** Comparison of demographic between OCD and HC

Variable	OCD	HC	t/×2	*P* value
Male	20(50%)	20(52.6%)	0.054	0.816
Mean age	16.25 ± 0.87	16.24 ± 0.59	0.079	0.938
BMI (kg/m2)	20.39 ± 3.28	20.77 ± 2.36	−0.592	0.555
Years of education	10.80 ± 1.04	10.61 ± 0.86	0.904	0.369
YBOCS score	24.23 ± 5.45	1.71 ± 2.19	23.716	0.000**

***:*P* < 0.001; OCD: Obsessive Compulsive Disorder, HC: Healthy Control, BMI: Body Mass Index, YBOCS: Yale Brown Obsessive Compulsive Scale

### Comparison of facial radial lines between OCD and HC

Lower lip height in OCD patients was significantly lower than that in HC (Cohen’s *d* = 0.54, Table 4). The difference related to lower lip height was significant in male participants (Cohen’s *d* = 0.77) but not in female participants (Cohen’s *d* = 0.29). No significant differences were observed in the other 20 facial diameters (all *P* > 0.05).

**Table 4:** Comparison of lower lip height ($\overline{\mathrm{x}}$±s)

	n	Lower lip height	t	P value
			−2.415	0.018*
OCD	40	7.68 ± 1.69		
HC	38	8.56 ± 1.52		
Male			−2.427	0.020^†^
OCD	20	7.86 ± 1.83		
HC	20	9.12 ± 1.44		
Female			−0.890	0.379
OCD	20	7.51 ± 1.56		
HC	18	7.94 ± 1.39		

*:P < 0.05, ^†^:P < 0.025; OCD: Obsessive Compulsive Disorder, HC: Healthy Control

### Comparison of facial angle between OCD and HC groups

The nasolabial angle in female OCD patients was significantly lower than that in the control group (Cohen’s *d* = 0.84, Table 5). No significant differences were observed in the whole sample and in other facial angles (all P > 0.05).

**Table 5:** Comparison of nasolabial angle ($\overline{\mathrm{x}}$±s)

	n	Nasolabial angle	t	P value
			−1.363	0.177
OCD	40	93.69 ± 9.20		
Control	38	97.06 ± 12.51		
Male			0.367	0.716
OCD	20	94.82 ± 10.06		
Control	20	93.50 ± 10.59		
Female			−2.618	0.013^†^
OCD	20	92.56 ± 8.35		
Control	18	101.03 ± 11.48		

^†^:P < 0.025; OCD: Obsessive Compulsive Disorder, HC: Healthy Control

### Relationship between facial and clinical features in OCD and HC groups

Lower lip height in the OCD group was positively correlated with the neutralizing dimension (r = 0.401, *P* < 0.05). No correlation was observed with other symptom dimensions (Table 6). Lower lip height in male OCD patients was not correlated with the OCI-CV. Similarly, nasolabial angle in female OCD patients was not correlated with the OCI-CV.

**Table 6:** Correlation between lower lip height and clinical features in OCD and HC

OCI-CV	r value	P value
Total score for OCD symptoms	0.252	0.116
Obsessing	− 0.009	0.957
Hoarding	0.055	0.736
Washing	0.057	0.729
Ordering	−0.263	0.101
Neutralizing	0.401	0.010*
Doubting/ Checking	0.042	0.797

*:P < 0.05; OCD: Obsessive Compulsive Disorder, HC: Healthy Control, OCI-CV: Obsessive Compulsive Inventory-Child Version

## Discussion

In this study, we compared the facial morphology of adolescent OCD patients with that of HC. We observed that the lower lip height of OCD patients was significantly lower than that of HC, suggestive of thinner lower lips in OCD patients. The lower lip redness of male OCD patients was significantly lower than that of male HC, indicating that the lower lips of male OCD patients were thinner. The nasolabial angle of female OCD patients was significantly smaller than that of female HC. Nasolabial angle reflects morphological changes of the nose and upper lip. An upturned nose tip or protruding upper lip will lead to a smaller angle. The facial morphology differences of the aforementioned OCD patients were concentrated in the nose and lips [20]. The facial features of OCD patients may originate from the facial brain development that occurs in the first and second trimesters of pregnancy. Facial brain morphogenesis is a major midline process [29]. The abnormal facial morphology of OCD patients centered in the nose and lips, which are located along the facial midline. During embryonic development, the mid-face is filled with cranial neural crest cells, which modulate forebrain development [20]. The nose and lips develop from the frontal nasal process of the embryonic primordia. According to the embryonic model, specific brain regions and areas of craniofacial abnormalities are linked. This suggested that abnormal frontal nasal derivatives in OCD may be related to the front and back of embryonic brain regions.

The frontal and posterior diencephalon correspond to the frontal and thalamic regions, respectively [22]. This is consistent with structural abnormalities of the putamen/globus pallidus, thalamus, prefrontal cortex, and caudate nucleus in children with OCD [31]. Previous studies found that patients with mental disorders such as schizophrenia and bipolar disorder also had abnormal facial morphology with typically concentrated in the frontonasal area [32], which was same as OCD patients. Collectively, these results suggested potential shared biological basis of schizophrenia and OCD.

As mentioned above, we observed that the nasolabial angle of female OCD patients was smaller than that of HC. These results indicated that the nose tip was upturned, or upper lip was protruding, suggesting that female OCD patients had a higher degree of facial protrusion. During the development of the facial and brain, the face grows forward faster than the brain; this difference in growth may underpin the decrease in forebrain growth and protrusion of the anterior midface [33]. Based on the correspondence between embryonic facial and brain development, it is speculated that frontal cortical development in female OCD patients was diminished [33]. This result is consistent with the neurobiological basis of abnormal frontal cortical development in children with OCD [34]. However, this result was not observed in male OCD patients, possibly due to the small sample size and limited measurement accuracy of the two-dimensional photographic facial measurement method. Future studies should expand the sample size and employ three-dimensional face laser-scanning technology to clarify these issues.

However, a lot of other facial diameters (for example minimum forehead width and face width) and facial angles (for example full surface coign, upper lip and chin process) were the same in OCD patients and HC [20, 31]. The influencing factors for facial morphological features are multidimensional and the effect of obsessive symptoms may be small. Generally speaking, the differences in facial features depend more on heredity. Interestingly, a 75% heritability of facial morphological features was found. The huge impact from heredity is difficult to change by the environment and personal traits like obsessive symptoms [35]. In addition, the non-significant facial features may be insensitive features to obsessive symptoms. Lower lip height and facial angles that were found to be significant indicators in the current study could be facial features specific to obsessive symptoms, which addresses the significance of our findings.

The present study has significant implications for future studies. Abnormal facial morphology can reflect abnormal brain morphology and is easier to measure than brain structure. Facial morphological variation can be used for in-body functional research [36]. Genetic studies of the face have reported that the *DCHS2* gene is associated with nasolabial angle and nose protrusion. Further, lip morphology is related to genes ACAD9, HOXD cluster, FREM1, and RAB7A [37]. Based on this, it is speculated that the above-mentioned genes may be related to OCD candidate genes, which need to be further verified in future studies.

In this study, correlational analysis between facial and clinical features of adolescents with OCD or HC revealed that the lower lip redness of facial features was positively correlated with neutralizing. This suggests that OCD patients with different symptom dimensions may have different facial features. Due to the high heritability of facial morphology and corresponding genetic underpinnings, it is speculated that the dimension of neutralizing symptoms may be more hereditary and may be modulated by the relevant genes. Completion of the facial genetic maps, advancements in three-dimensional facial imaging quantitative analysis, and application of facial recognition technology in disease diagnosis [24], may lead to pathogenic genes underpinning OCD being identified based on facial morphological variation.

This study adopted the method of measuring participants’ facial features in frontal and lateral two-dimensional photographs. This method only permitted two-dimensional, but not three-dimensional, structural features of the face to be obtained. This may have overlooked minor variations in facial features. Future research should employ facial 3D laser scanning photographic technology or artificial intelligence facial recognition technology to collect images and use computer deep-learning technology to analyze facial feature images to obtain more facial variation information [33]. Further, the use of facial features as neurodevelopmental indicators did not verify the relationship between facial and brain morphology. In the future, this approach can be combined with brain magnetic resonance imaging to improve the extrapolation of results. Considering MPAs also occur in children with neurodevelopmental disorders such as ADHD, Tourette and other tic disorders, the lack of information related to the comorbidity may confound the relationship between obsessive symptoms and facial features. In addition, we identified facial feature-related variation in adolescents with OCD and used human facial genetic maps to speculate on susceptibility genes that were associated with OCD, but causative mechanisms could not be verified. Animal models or in vivo studies should be performed in the future to clarify the relationship between related genes and OCD. The cross-sectional design limited our ability to infer temporal and potential causal effects between facial characteristics and OCD in adolescents. The small sample size of the current study needs to be acknowledged. The gender differences related to lower lip redness and nasolabial angle were easily affected by the small sample size, leading to estimation bias. In addition, the small sample size suggested a population with poor diversity which may limit the generalizability of our findings. Future research should use larger sample sizes with longitudinal or cross-lag designs to provide insight into causal mechanisms.

## Conclusions

In conclusion, this study identified a correlation between facial features and OCD. Male OCD patients had a thinner lower lip and female OCD patients had smaller nasolabial angles. The facial features of adolescents with OCD were positively correlated with lower lip redness and neutralizing.

## Data Availability

The datasets used and analyzed during the current study are available from the corresponding author on reasonable request.

## Abbreviations

OCI-CV: Child Obsessive Compulsive Scale
MPAs: Minor physical anomalies
OCD: obsessive-compulsive disorder
HC: healthy controls
YBOCS: The Yale Brown Obsessive Compulsive Scale
